# A case of malignant catatonia with idiopathic pulmonary arterial hypertension treated by electroconvulsive therapy

**DOI:** 10.1186/s12888-016-0835-4

**Published:** 2016-05-06

**Authors:** Mizue Hobo, Akihito Uezato, Mitsunori Nishiyama, Mayumi Suzuki, Jiro Kurata, Koshi Makita, Naoki Yamamoto, Toru Nishikawa

**Affiliations:** Department of Psychiatry and Behavioral Sciences, Graduate School of Medical and Dental Sciences, Tokyo Medical and Dental University, 1-5-45 Yushima, Bunkyo-ku, Tokyo, 113-8519 Japan; Clinical Center for Pleasant Sleep, Medical Hospital, Tokyo Medical and Dental University, Tokyo, Japan; Department of Pediatrics, Graduate School of Medical and Dental Sciences, Tokyo Medical and Dental University, Tokyo, Japan; Department of Anesthesiology, Graduate School of Medical and Dental Sciences, Tokyo Medical and Dental University, Tokyo, Japan

**Keywords:** Electroconvulsive therapy, Idiopathic pulmonary arterial hypertension, Malignant catatonia, Pulmonary hypertensive crisis, Remifentanil

## Abstract

**Background:**

Idiopathic pulmonary arterial hypertension (IPAH) is a progressive and fatal cardiovascular disease if left untreated. In patients with IPAH with psychiatric illness or other complications, careful attention is required when administering medical therapies that may affect their hemodynamics. Patients suffering from IPAH who undergo anesthesia and surgery have a high mortality and morbidity rate. We describe the treatment of intractable psychiatric symptoms with electroconvulsive therapy (ECT) in a patient with IPAH.

**Case presentation:**

A 23-year-old woman with IPAH and type I diabetes mellitus (DM) presented with malignant catatonia. Her heart function was classified as New York Heart Association (NYHA) class III. She required a rapid cure and ECT due to various psychiatric symptoms resistant to conventional medications. Pulmonary hypertensive (PH) crisis is the most concerning complication that can be induced by the sympathetic stimulation of ECT. To avoid PH crisis, we administered oxygen using a laryngeal mask and administered remifentanil for anesthesia. We also prepared standby nitric oxide for possible PH crisis, although it was ultimately not needed. With 14 ECT sessions, her malignant catatonia was ameliorated without physical complications.

**Conclusion:**

ECT is an acceptable option for the treatment of medication-refractory psychiatric disturbances in patients with IPAH, provided careful management is assured to prevent or address complications.

## Background

Patients with idiopathic pulmonary arterial hypertension (IPAH) who undergo anesthesia and surgery have a high mortality and morbidity rate [1]. Careful attention is required when administering medications or therapies that may affect the patients’ hemodynamics. Regardless, malignant catatonia is a life-threatening psychiatric condition that needs systemic care and a rapid cure. We treated malignant catatonia in a patient with IPAH who eventually required electroconvulsive therapy (ECT) that could have affected her hemodynamics. This report describes the first case of malignant catatonia with IPAH treated by ECT and the risk-reducing procedures we used to prevent physical complications.

## Case presentation

A 23-year-old woman with a history of IPAH presented to our service with malignant catatonia. She was diagnosed with IPAH at the age of 8 due to her high mean pulmonary arterial pressure (PAP) of 50 mmHg (a diagnosis of IPAH is made if PAP is >25 mmHg according to the World Symposium on Primary Pulmonary Hypertension 1998). Because her IPAH progressed despite treatment with beraprost and home oxygen therapy, she began to receive 24-hour infusions of epoprostenol (prostaglandin I_2_) at the age of 12. Epoprostenol was temporarily effective to improve pulmonary circulation, but when she was 23 years old, her heart function deteriorated to the New York Heart Association (NYHA) class III [2]. At age 16, the patient was also diagnosed with insulin-resistant diabetes mellitus (DM) and was started on insulin injections.

The patient’s significant medical history also included her psychiatric illnesses. She was hospitalized at the ages of 20 and 21 because of attempted suicide under depressed mood with psychomotor excitement and psychosis. Therefore, she had been treated with antidepressants and antipsychotics for psychotic depression, but she was later re-diagnosed with schizophrenia during the present hospitalization.

At the age of 23 years, the patient became non-compliant with psychiatric medications. One month later, she became agitated and began muttering and then was gradually rendered mute and cataleptic. Subsequently, she was hospitalized at our psychiatric ward because of a substuporous state. Despite continuing her cardiovascular drugs, she was physically unstable with tachycardia, hypertension, a fluctuating blood glucose level ranging from 50 to 400 mg/dL, and hyperthermia (up to 40.0 °C). The findings of cardiac examination on admission are shown in Table 1. The medications used to treat these somatic problems were oral digitalis (0.125 mg/d), spironolactone (25 mg/d), warfarin (2 mg/d), furosemide (40 mg/d), epoprostenol, and subcutaneous insulin injections.

**Table 1:** Cardiac examination findings on admission

		Current case	Normal values
	systolic	74	15–28
PAP (mmHg)	diastolic	27	5–16
	mean	45	10–22
CI (L/min/m2)		3.21	2.3–4.2
BNP (pg/ml)		133.4	<20
CR (%)		64	50
EF (%)		53	55–80
Oxygen Saturation (%)		90	96–99

*PAP* pulmonary arterial pressure, *CI* cardiac index, *BNP* brain natriuretic peptide, *CR* cardiothoracic ratio, *EF* ejection fraction

**Fig. 1:**
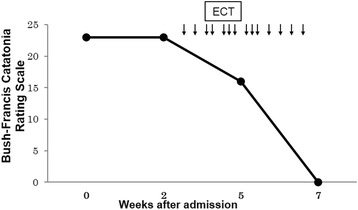
Time course of catatonia assessed by Bush- Francis Catatonia Rating Scale Catatonia is rated at 23 on admission, and symptoms are alleviated in response to ECT sessions. The down arrows indicate each ECT session

Head magnetic resonance imaging (MRI), electroencephalography (EEG), and cerebrospinal fluid (CSF) analysis were unremarkable. We determined that the most likely diagnosis for her condition, including delirium, mutism, catalepsy, hyperpyrexia, posturing, and autonomic instability, was malignant catatonia according to the criteria of Fink [3]. The score on the Bush-Francis Catatonia Rating Scale [4] was 23 on admission (Fig. 1). Lorazepam (given orally, 3 mg per day) alleviated the substuporous state, but she developed Capgras’ syndrome and persecutory delusions and exhibited agitation. Levomepromazine (50 mg/day) and aripiprazole (24 mg/day) were not effective for these symptoms. Because her autonomous and hemodynamic instability was potentially life threatening, we decided to introduce ECT using a square wave-generating instrument (Thymatron System IV; Somatics, LLC, Lake Bluff, IL, USA) after obtaining informed consent from her family. To monitor for potential pulmonary hypertensive (PH) crisis, which is the most serious complication of ECT, a pediatric cardiologist and medical engineer carefully observed her cardiopulmonary status during all ECT sessions. Endotracheal intubation was performed, and standby nitric oxide (NO) inhalation was prepared in case of PH crisis secondary to dilating pulmonary arteries. Moreover, during each ECT procedure, epoprostenol was continuously infused to prevent PH, and a combination of propofol and remifentanil was used to induce anesthesia. Remifentanil was chosen because it is a short acting opioid with strong anti-nociceptive features and was expected to stabilize blood pressure. Propofol was injected intravenously using a target-controlled infusion (TCI) at blood concentrations between 3 and 4 mcg/mL, which can keep most patients unconscious, and remifentanil was infused at 0.5-1.0 mg/h, followed by rocuronium (0.8 mg/kg) to relax the muscles. TCI is a drug infusion technique that combines a real-time pharmacokinetic model with an infusion pump and allows the administration and maintenance of a constant blood concentration of drug.

The ECT electrodes were positioned bilaterally through all sessions. For the first ECT session, a stimulus dose of 25 % was administered, and seizures were observed for 59 s by electromyography (EMG) and 69 s by EEG. Though the patient was expectedly tachycardic during the intubation procedure and after convulsion, her blood pressure was kept within the expected range. Therefore, we speculated that pulmonary artery constriction did not occur and that NO was not needed. Because she complained of intense throat pain, we used a laryngeal mask instead of intubation for subsequent ECT sessions. This was determined after discussing with the anesthesiologists who assured a laryngeal mask would be sufficient for NO inhalation and oxygenation. Moreover, we speculated she would need more than 10 ECT sessions but were concerned she would not tolerate repeated intubation. As the ECT sessions were repeated, the symptoms of malignant catatonia, such as tachycardia, hypertension, fluctuating blood glucose and hyperthermia, were gradually alleviated, and these parameters were generally within the normal ranges after the ninth session. Eventually, we completed 14 ECT sessions with an average stimulus dose of 56.6 %, which is equivalent to 285 mC. The average seizure durations were 35.9 s on EMG and 39.3 s on EEG. The Bush-Francis Catatonia Rating Scale score decreased to zero after the last ECT session (Fig. 1). Upon completion of the ECT, her mental state returned to her baseline. She was discharged from the hospital 93 days after admission. Follow-up was continued on an outpatient basis with the patient in remission with the oral administration of aripiprazole (30 mg/day), valproic acid (600 mg/day), lorazepam (1 mg/day), zotepine (150 mg/day), digitalis (0.125 mg/day), spironolactone (25 mg/day), warfarin (2 mg/day), and furosemide (40 mg/day) in addition to epoprostenol and insulin therapies.

The patient and/or her family had shared their perspectives on the treatments she received. In particular, when the patient was considered to be not competent in terms of understanding her medical condition, the treatment team gave her family a full explanation regarding her condition and treatment options, including ECT and medication treatments. Then, when the patient recovered well enough to be competent, the same process was repeated to obtain her informed consent.

## Discussion

To our knowledge, this is the first report to describe ECT application in a patient with IPAH. We administered ECT and treated malignant catatonia complicated by IPAH. ECT is considered to be a first-line treatment for malignant catatonia [5, 6]. However, patients with IPAH who undergo anesthesia and surgery have a high mortality and morbidity rate. Indeed, it has recently been reported in a series of patients with mild to moderate pulmonary hypertension during non-cardiothoracic non-obstetric surgery that 29 % of the patients had complications and that four of 28 patients died [7].

In our patient, PH crisis was the most potential concerning complication of ECT [8]. PH crisis is a consequence of constricted pulmonary arteries, which is induced by factors such as hypoxia or sympathetic stimulation in patients with pulmonary hypertension. Rapidly constricting pulmonary arteries in the PH crisis lead to right heart failure and severe hypoxia [9]. These risks can be effectively reduced based upon our clinical experiences and based on published reports in the field of IPAH and PH crisis [10]. First, we utilized endotracheal intubation or a laryngeal mask in order to provide sufficient oxygenation and to prevent hypercapnia. Second, we selected remifentanil for anesthesia, because it has strong anti-nociceptive features and was expected to stabilize blood pressure [11]. Third, we had an NO appliance available in case of PH crisis.

## Conclusion

ECT is an acceptable option for treatment of intractable psychiatric symptoms in a patient with IPAH, provided patients are managed carefully to prevent or address potential complications.

### Ethics approval and consent to participate

Not applicable.

### Consent

Written informed consent was obtained from the patient for publication of this case report. A copy of the written consent is available for review by the Editor of this journal.

### Availability of data and materials

All the data supporting our findings will be shared upon request.

## Abbreviations

CSF: cerebrospinal fluid
DM: diabetes mellitus
ECT: electroconvulsive therapy
EEG: electroencephalography
IPAH: idiopathic pulmonary arterial hypertension
MRI: magnetic resonance imaging
NO: nitric oxide
NYHA: New York Heart Association
PAP: pulmonary arterial pressure
PH: pulmonary hypertensive
TCI: target-controlled infusion
